# Upregulated WEE1 protects endothelial cells of colorectal cancer liver metastases

**DOI:** 10.18632/oncotarget.15039

**Published:** 2017-02-02

**Authors:** Peter J. Webster, Anna T. Littlejohns, Hannah J. Gaunt, Richard S. Young, Baptiste Rode, Judith E. Ritchie, Lucy F. Stead, Sally Harrison, Alastair Droop, Heather L. Martin, Darren C. Tomlinson, Adam J. Hyman, Hollie L. Appleby, Sally Boxall, Alexander F. Bruns, Jing Li, Raj K. Prasad, J. Peter A. Lodge, Dermot A. Burke, David J. Beech

**Affiliations:** ^1^ School of Medicine, University of Leeds, Leeds LS2 9JT, UK; ^2^ School of Biological Sciences, University of Leeds, Leeds LS2 9JT, UK; ^3^ Department of Hepatobiliary and Transplant Surgery, St. James's University Hospital, Leeds LS9 7TF, UK; ^4^ Department of Colorectal Surgery, St. James's University Hospital, Leeds LS9 7TF, UK; ^5^ MRC Medical Bioinformatics Centre, University of Leeds, Leeds LS2 9NL, UK

**Keywords:** checkpoint kinase, angiogenesis, colorectal cancer, DNA damage, endothelial cell

## Abstract

Surgical resection of colorectal cancer liver metastases (CLM) can be curative, yet 80% of patients are unsuitable for this treatment. As angiogenesis is a determinant of CLM progression we isolated endothelial cells from CLM and sought a mechanism which is upregulated, essential for angiogenic properties of these cells and relevant to emerging therapeutic options. Matched CLM endothelial cells (CLMECs) and endothelial cells of normal adjacent liver (LiECs) were superficially similar but transcriptome sequencing revealed molecular differences, one of which was unexpected upregulation and functional significance of the checkpoint kinase WEE1. Western blotting confirmed that WEE1 protein was upregulated in CLMECs. Knockdown of WEE1 by targeted short interfering RNA or the WEE1 inhibitor AZD1775 suppressed proliferation and migration of CLMECs. Investigation of the underlying mechanism suggested induction of double-stranded DNA breaks due to nucleotide shortage which then led to caspase 3-dependent apoptosis. The implication for CLMEC tube formation was striking with AZD1775 inhibiting tube branch points by 83%. WEE1 inhibitors might therefore be a therapeutic option for CLM and could be considered more broadly as anti-angiogenic agents in cancer treatment.

## INTRODUCTION

Colorectal cancer is a global health problem with an estimated 1.4 million cases and 693,900 deaths occurring per year [1]. Liver metastases remain the major cause of death in these patients. Approximately 25% of colorectal cancer patients will present with synchronous liver metastases and up to 50% develop metachronous liver metastases [2]. Hepatic resection remains the only curative treatment for colorectal cancer liver metastases (CLM) with 5-year survival rates of up to 58% when combined with neo-adjuvant or adjuvant chemotherapy [3]. However, only 20% of patients with CLM are suitable for curative liver surgery at the time of diagnosis [4]. For the remaining patients, chemotherapy is largely palliative with a median survival of 21 months [5].

A particular feature of CLM are micrometastatic foci which cause endothelial cells to migrate, proliferate and invade to form neo-vessels enabling tumour growth and progression [6]. The use of agents that target endothelial signaling has had success. Bevacizumab (Avastin**^®^**) is a recombinant humanized monoclonal antibody that inhibits Vascular Endothelial Growth Factor A and has clinical benefit when used in addition to combination chemotherapy for the treatment of metastatic colorectal cancer [7]. Unfortunately resistance to therapy can become a problem [8], but even so, the observed clinical effect justifies continued efforts to target the CLM endothelial cells. A major brake on developing therapies targeted to these cells is the lack of specific knowledge about their properties and molecular mechanisms. In an effort to overcome this limitation we have focused on studies of CLM endothelial cells, comparing them to endothelial cells from adjacent normal liver of the same patient. This approach led us to WEE1.

WEE1 is a protein kinase that regulates cell entry into mitosis at the G2/M checkpoint through the activity of cyclin-dependent kinase 1 (CDK1). In response to DNA damage, WEE1 inactivates CDK1 through an inhibitory phosphorylation of its tyrosine 15 residue (pCDK1-Y15) [9]. This results in G2 arrest and allows DNA damage to be repaired. Initial pre-clinical studies reported that WEE1 inhibition in combination with DNA damaging agents forced cancer cells into premature mitosis with lethal unrepaired DNA damage resulting in cell death [10–12]. Indeed this has resulted in a number of Phase II clinical trials involving AZD1775, a WEE1 inhibitor, in combination with DNA damaging agents in a range of cancers (http://www.clinicaltrials.gov). More recently, AZD1775 has been shown to cause excess origin firing because of increased active CDK1 (as a result of WEE1 inhibition), leading to nucleotide exhaustion and double-stranded DNA breaks [13]. This has been supported by several studies showing single agent activity for AZD1775 in the absence of DNA damaging agents in cancer cells [14–16]. There have not however been studies of WEE1 in endothelial cells.

## RESULTS

### CD31-positive cells isolated from CLM are endothelial cells

**Figure 1:**
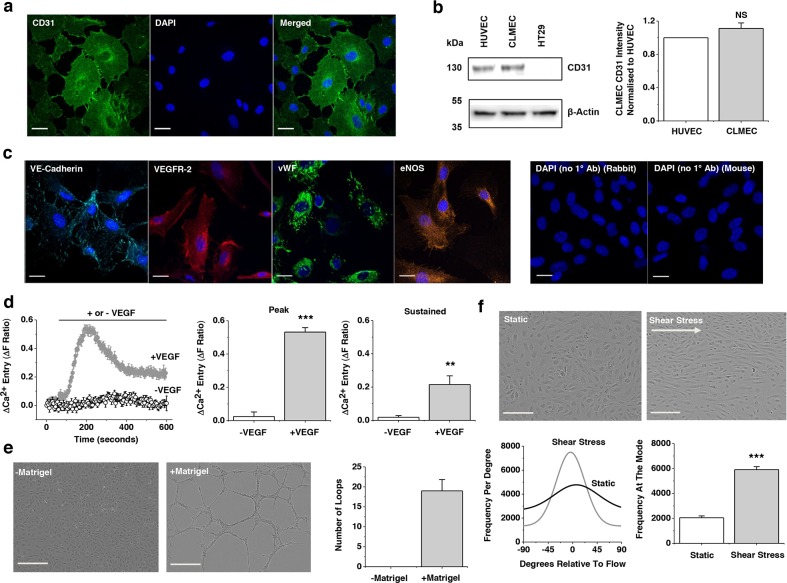
Colorectal cancer liver metastases endothelial cells (CLMECs) are superficially similar to other types of endothelial cells **a**. Immunofluorescence images of CLMECs stained with anti-CD31 antibody (green) and DAPI to label nuclei (blue). Scale bars 20 μm. **b**. On the left, Western blot labelled with anti-CD31 and anti-β-Actin antibodies for CLMECs, human umbilical vein endothelial cells (HUVECs) and a human colorectal adenocarcinoma cell line (HT29). On the right, quantification of the CD31 band intensity relative to β-Actin and HUVEC (n=3 each). **c**. Immunofluorescence images of CLMECs stained with anti-VE-Cadherin (turquoise), anti-Vascular Endothelial Growth Factor Receptor 2 (VEGFR-2, red), anti-von Willebrand Factor (vWF, green) and anti-endothelial Nitric Oxide Synthase (eNOS, orange). In each image nuclei were labelled with DAPI (blue). Control images in the absence of the primary antibodies are shown on the right. Scale bars 20 μm. **d**. Intracellular Ca^2+^ measurement data from CLMECs. On the left, averaged responses to 30 ng.mL^−1^ VEGF across multiple wells of a 96-well plate compared to control (N=5 wells each). On the right, mean data for the peak (200 s) and sustained (600 s) responses to VEGF of the type exemplified on the left (n=3, N=15). **e**. On the left, images of CLMECs in the absence and presence Matrigel®. Scale bars 250 μm. On the right, quantification of the number of complete loops seen in images of the type shown on the left (n=4, N=12). **f**. Top panels: Images of CLMECs in static condition and after shear stress. Scale bars 80 μm. Bottom left: Example orientation analysis for the images shown at the top. Bottom right: Mean data for the analysis shown on the left (n=3, N=18).

Cells isolated from CLM using anti-CD31 beads continued to display expression of the CD31 endothelial marker protein after low-passage cell culture (Figure 1a). The commonly studied human umbilical vein endothelial cells (HUVECs) expressed CD31 similarly whereas the colorectal cancer cell line HT29 did not (Figure 1b). The CLM cells stained positive for other endothelial cell markers including VE-Cadherin, Vascular Endothelial Growth Factor 2 (VEGFR-2), von Willebrand Factor (vWF) and endothelial Nitric Oxide Synthase (eNOS) (Figure 1c). Also as expected for endothelial cells they responded with an intracellular Ca^2+^ elevation upon application of Vascular Endothelial Growth Factor (VEGF) (Figure 1d) and formed lattice-like structures on the artificial extracellular matrix Matrigel^®^ (Figure 1e). Moreover, they aligned to the direction of shear stress, another endothelial cell characteristic (Figure 1f). The data suggest that the cells can be classified as endothelial. Henceforth they are referred to as CLMECs. Using similar techniques we isolated and characterised endothelial cells from normal healthy liver adjacent to CLM and refer to these cells as LiECs.

### WEE1 is upregulated and functionally significant in CLMECs

**Figure 2:**
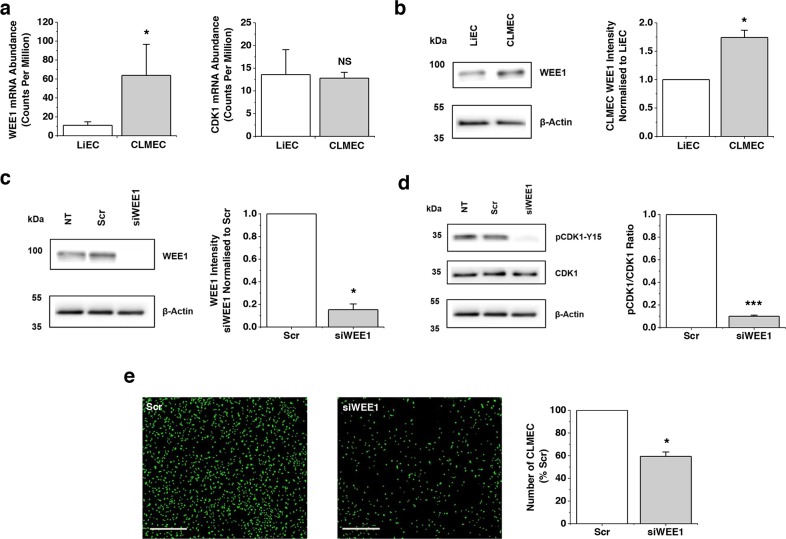
WEE1 is upregulated and functional in CLMECs **a**. WEE1 (left) and CDK1 (right) transcript abundances in CLMECs compared to matched liver endothelial cells (LiECs) (n=3 each). The data are from RNAseq analysis which is provided in detail in Supplementary Data File 1. **b**. On the left, Western blot labelled with anti-WEE1 and anti-β-Actin antibodies for LiECs and CLMECs. On the right, quantification of the CLMEC WEE1 band intensity relative to β-Actin and LiEC (n=3 each). **c**. On the left, Western blot labelled with anti-WEE1 and anti-β-Actin antibodies for non-transfected (NT) CLMECs and CLMECs transfected with scrambled siRNA (Scr) or WEE1-targeted siRNA (siWEE1). On the right, quantification of the WEE1 band intensity for the siWEE1 group normalised to the Scr group (n=3 each). **d**. On the left, Western blot labelled with anti-pCDK1-Y15, anti-CDK1 and anti-β-Actin antibodies for non-transfected (NT) CLMECs and CLMECs transfected with scrambled siRNA (Scr) or WEE1-targeted siRNA (siWEE1). On the right, quantification of the pCDK1-Y15 band intensity divided by the CDK1 intensity (n=3 each). **e**. On the left, fluorescence images of CLMECs 48 hr after transfection with scrambled (Scr) or WEE1 siRNA (siWEE1). Fluorescence was from cell nuclei stained with Vybrant® Dye Cycle™ (green). Scale bars 400 μm. On the right, normalised quantification of the images from independent repeats (n=3, N=18).

Although CLMECs and LiECs were superficially similar, RNA sequencing of paired CLMECs and LiECs from the same patient revealed differentially expressed transcripts (Supplementary Data File 1, Supplementary Figures 1, 2). Here we focused on the upregulated transcript which encodes WEE1 (Figure 2a). Transcript encoding its target protein CDK1 was, by contrast, unchanged (Figure 2a). Importantly, Western blotting showed that WEE1 protein was, like WEE1 mRNA, upregulated (Figure 2b). Knockdown of WEE1 was achieved using targeted short interfering RNA (Figure 2c). As expected it resulted in strong reduction in pCDK1-Y15 (Figure 2d). Functionally, knockdown of WEE1 inhibited proliferation of CLMECs by 41% (Figure 2e). The data suggest that WEE1 is upregulated in CLMECs and that its knockdown impairs CLMEC proliferation.

### Small-molecule inhibition of WEE1 has preferential effects on CLMECs

**Figure 3:**
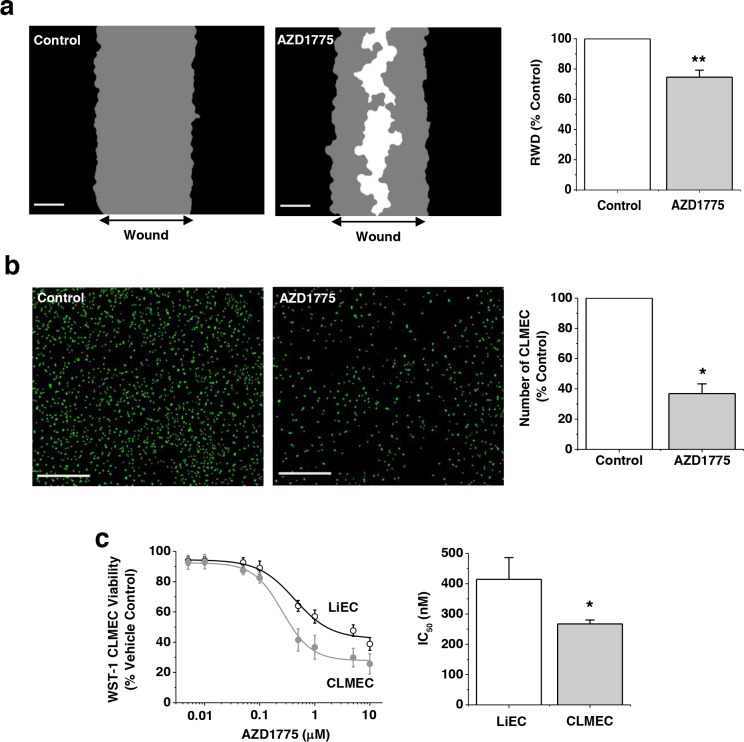
Small-molecule inhibition of WEE1 has preferential effect on CLMECs **a**. On the left, example linear wound mask images after 24 hr CLMEC migration in vehicle control (Control) or 1 μM AZD1775. Black represents cells outside the linear wound (top to bottom), grey - cells which had migrated into the wound, and white - no cells. Scale bars 200 μm. On the right, mean data from images of the type shown on the left. Relative wound density (RWD) in the presence of AZD1775 is presented as a percentage of Control (n=3, N=9). **b**. On the left, fluorescence images of CLMECs labelled with Vybrant® Dye Cycle™ (green) 48 hr after incubation in vehicle control or 1 μM AZD1775. Scale bars 400 μm. On the right, normalised quantification of the images from independent repeats (n=6, N=18). **c**. On the left, mean data for CLMEC and LiEC viability measured using WST-1 reagent after treatment with AZD1775 at the indicated concentrations and plotted as percentages of the vehicle control (n=6, N=18 each). On the right, IC_50_s from the fitted Hill equations shown on the left.

If upregulated WEE1 is important for CLMECs, they should be sensitive to the small-molecule WEE1 inhibitor AZD1775. We therefore tested AZD1775. AZD1775 (1 μM) inhibited CLMEC migration by 20% and proliferation by 63% (Figure 3a, 3b). There were no differences in migration or proliferation rates between matched, untreated LiECs and CLMECs (Supplementary Figure 3). Concentration-response curves were constructed for the effect of AZD1775 on cell proliferation. The derived IC_50_ for AZD1775 against CLMEC proliferation was 267 nM, significantly less than for LiECs (Figure 3c). These data are consistent with greater functional importance of WEE1 in CLMECs compared with LiECs.

### WEE1 protects against double-stranded DNA breaks in CLMECs

**Figure 4:**
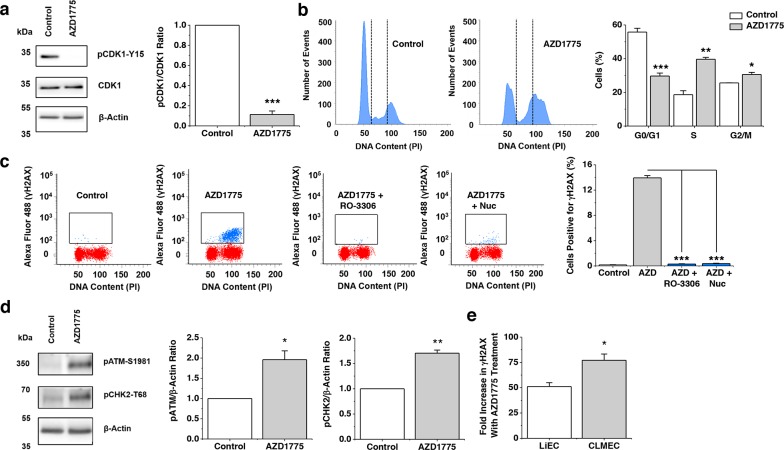
WEE1 protects against double-stranded DNA breaks **a**. On the left, Western blot labelled with anti-pCDK1-Y15, anti-CDK1 and anti-β-Actin antibodies for CLMECs treated for 24 hr with vehicle control or 1 μM AZD1775. On the right, quantification of the pCDK1-Y15 band intensity divided by the CDK1 intensity (n=3 each). **b**. On the left, example flow cytometry data for CLMECs 24 hr after treatment with 1 μM AZD1775 or vehicle control. The vertical dotted lines separate different phases of the cell cycle. On the right, the mean percentage of cells in G0/G1, S and G2/M phases (n=3 each). AZD1775 data are compared with Controls for each phase. **c**. On the left, four example flow cytometry dot plots for unlabelled CLMECs (red) and CLMECs labelled with anti-γH2AX antibody (blue). The four conditions were vehicle control, 1 μM AZD1775, 1 μM AZD1775 + 10 μM RO-3306, and 1 μM AZD1775 + exogenous nucleosides (Nuc) (EmbryoMax^®^, 1:5 dilution) for 24 hr. On the right, mean data for the four groups (n=3 each). **d**. On the left, Western blot labelled with anti-pATM-S1981, anti-pCHK2-T68 and anti-β-Actin antibodies for CLMECs treated for 24 hr with vehicle control or 1 μM AZD1775. On the right, quantification of the pATM-S1981 and pCHK2-T68 band intensity divided by the β-Actin intensity (n=3 each). **e**. Mean data for the percentage fold increase in γH2AX positive LiECs and CLMECs with AZD1775 treatment (n=3 each).

To investigate the mechanism by which upregulated WEE1 protects CLMECs we considered ideas from cancer cell studies where AZD1775 has been found to induce double-stranded DNA breaks. In CLMECs, AZD1775 reduced pCDK1-Y15 and caused significantly more CLMECs to be in S and G2/M phase (Figure 4a, 4b). Flow cytometry was then used to gate for CLMECs which were positive for histone 2A phosphorylation (γH2AX), a marker of double-stranded DNA breaks. Strikingly AZD1775 caused a 77-fold increase in γH2AX (Figure 4c). Consistent with the effect being caused by increased CDK1 activity (because of reduced inhibitory phosphorylation of CDK1) it was prevented by the CDK1 inhibitor R0-3306 (Figure 4c). Also consistent with this mechanism was the protective effect of exogenous nucleosides which compensated nucleotide exhaustion caused by excessive CDK1 activity (Figure 4c). As would be expected with double-stranded DNA breaks, AZD1775 treatment activated genotoxic stress pathways, with significant upregulation of phosphorylated-ATM and phosphorylated-CHK2 (Figure 4d). When compared with matched LiECs, AZD1775 caused significantly more double-stranded DNA breaks in CLMECs (Figure 4e). The data suggest that CLMECs use upregulated WEE1 to protect against double-stranded DNA breaks.

### WEE1 protects CLMECs against caspase-3-dependent apoptosis

**Figure 5:**
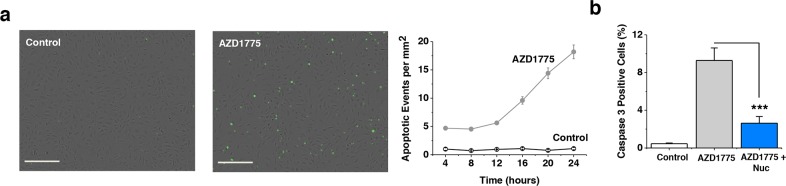
WEE1 protection against caspase-3-dependent apoptosis **a**. Images of fluorescence from caspase-3 activity indicator in CLMECs 24 hr after treatment with vehicle control or 1 μM AZD1775. Scale bars 200 μm. On the right, mean number of caspase-3 positive CLMECs per mm^2^ at the indicated time points after AZD1775 treatment or vehicle control (Control) (n=3 each). **b**. Mean data for experiments of the type shown in (a) after 24 hr treatment and including a 1 μM AZD1775 with exogenous nucleoside group (Nuc) (EmbryoMax^®^, 1:5 dilution) (n=3 each).

A potential consequence of double-stranded DNA breaks is increased apoptosis. Therefore we investigated a key mechanism of apoptosis - caspase-3-dependent apoptosis. WEE1 inhibition caused a 20-fold increase in apoptosis in CLMECs, suggesting that WEE1 constitutively protected the cells against apoptosis (Figure 5a). Exogenous nucleosides protected against the induced apoptosis, consistent with excessive CDK1 activity being its underlying cause (Figure 5b). The data suggest that WEE1-dependent protection against double-stranded DNA breaks is important for the avoidance of apoptosis in CLMECs.

### WEE1 protects CLMEC tube formation

**Figure 6:**
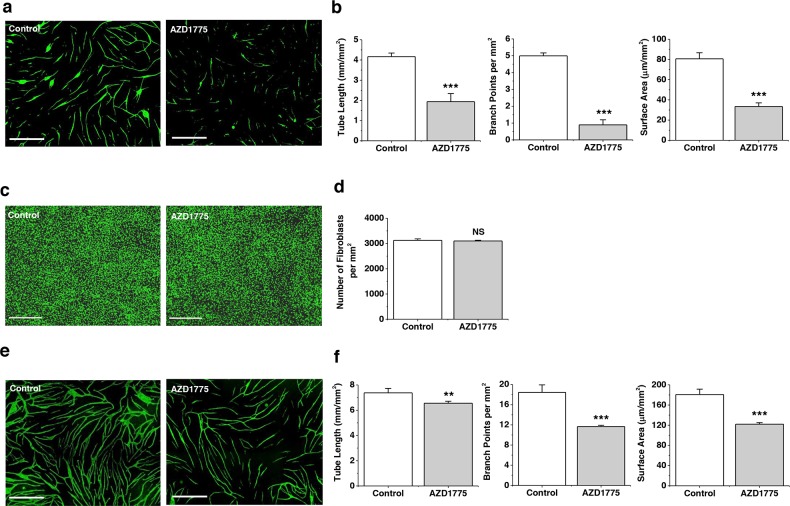
WEE1 protection of branching tube formation **a**. Fluorescence images of anti-CD31-labelled CLMECs (green) in tube formation on a bed of fibroblasts (the fibroblasts are not visible in the images). The co-cultures were treated daily for 5 days with 1 μM AZD1775 or its vehicle control (Control). Scale bars 800 μm. **b**. For experiments of the type exemplified in (a), mean data for tube length, number of branch points and tube surface area (n=3, N=9). **c**. Fluorescence images of confluent normal human dermal fibroblasts labelled with Vybrant® Dye Cycle™ (green) following incubation with 1 μM AZD1775 or its vehicle control (Control) for 5 days. Scale bars 400 μm. **d**. For experiments of the type exemplified in (c), mean number of fibroblasts per mm^2^ (n=3, N=9). **e**. Fluorescence images of anti-CD31-labelled HUVECs (green) in tube formation on a bed of fibroblasts (the fibroblasts are not visible in the images). The co-cultures were treated daily for 5 days with 1 μM AZD1775 or its vehicle control (Control). Scale bars 800 μm. **f**. For experiments of the type exemplified in (d), mean data for tube length, number of branch points and tube surface area (n=3, N=9).

CLM progression is thought to depend not on the existence of CLMECs *per se*, but on the capability of these cells to form tubes which deliver nutrients and remove waste products from the cancer cells and their supporting cells. In order to investigate this capability we developed a CLMEC co-culture assay in which the CLMECs formed tube-like structures on a bed of fibroblasts (Figure 6a). WEE1 inhibition had a striking inhibitory effect on these CLMEC tubes, reducing the total tube length, the total tube surface area and, most strongly, the number of branching points (Figure 6b). There was no effect on the integrity of the fibroblast bed (Figure 6c, 6d). The effect on tube formation was also evident when HUVECs were used in place of CLMECs but the amplitudes of these effects were smaller (Figure 6e, 6f). The data suggest a key role of WEE1 in protecting against caspase-3-dependent apoptosis which would otherwise suppress CLMEC tube formation.

## DISCUSSION

This study reveals previously unrecognised upregulation of WEE1 in CLMECs and proposes a mechanism by which it could facilitate CLM progression by protecting against apoptosis which restricts tube formation. The implication is that WEE1 inhibitors are likely to reduce the extent of tumour vascularisation independently of effects on tumour cells.

We suggest that WEE1 upregulation in CLMECs confers particular importance in the CLM context, but is there broader relevance? Endothelial cells are of course throughout the body and pivotal in health and disease. The relative functional importance of WEE1 in these contexts is unknown. Substantial impact in physiological blood vessels seems unlikely because the endothelial cells are normally quiescent. However, when there is cell cycle progression in vascular maintenance or wound-healing, roles of WEE1 might become apparent. A cell type commonly used to model such biology is the HUVEC. We detected WEE1 in these cells and observed effects of WEE1 inhibition (Supplementary Figure 4). The sensitivity to AZD1775 and functional relevance of WEE1 was nevertheless less than that seen in CLMECs. Results from Phase I clinical trials report no serious vascular complications [15] but this does not mean that such complications are lacking. Therefore designers of future trials should consider particular attention to assessments of wound-healing and cardiovascular parameters including those relating to risk of atherosclerotic disease.

The ability of AZD1775 to strongly inhibit CLMEC tube formation should be considered in the design of future clinical trials of this agent. Firstly, trials aimed at determining the impact on CLM as a mono or combination therapy are encouraged by our findings. Secondly, the design of such trials should take into consideration the potential for a direct anti-angiogenic effect and whether AZD1775 can only be used once patients have established CLM or whether there is a role for treatment after resection of the primary colorectal cancer to suppress or prevent metastatic disease. We speculate that upregulated WEE1 and its role in conserving branching tube formation in the face of apoptotic pressures could be relevant to other cancers in which angiogenesis is a key player. Therefore our observations might be considered in the design of future trials in which WEE1 inhibitors are considered as mono or combination therapies.

The stimulus and mechanism for WEE1 upregulation in CLMECs is unknown. Whatever the mechanism, it is sustained despite culturing of the CLMECs under physiological conditions. A potential stimulus for upregulation is hypoxia because mass spectrometry analysis of proteins in the MS-1 endothelial cell line found that hypoxia causes increased WEE1 mRNA and protein and phosphorylation of CDK1 [17].

WEE1 was not the most upregulated transcript identified by our RNAseq analysis (Supplementary Data File 1). We selected it because of its relevance to a newly emerging therapeutic strategy and because it was one of relatively few hits in a RNA interference screen against HUVEC proliferation (Supplementary Data File 2). The more highly upregulated transcripts may of course be attractive candidates for detailed investigation and some of these are established cancer biomarkers, tumour endothelial markers, proto-oncogenes and tumour suppressor genes, as well as genes important in colorectal cancer signaling and angiogenesis (Supplementary Figure 2).

In conclusion the data suggest the importance of WEE1 as an enabler of branching vascularisation in colorectal cancer liver metastases. It is a function which is likely to be important in tumour expansion and progression and therefore likely to be important in the clinical actions of WEE1 inhibitors such as AZD1775. The possibility to isolate and study endothelial cells from these metastases and compare them to endothelial cells of adjacent normal liver is attractive. It has revealed that the endothelial cells are superficially similar to normal endothelial cells but with a number of molecular differences which can be potentially exploited for therapeutic benefit.

## MATERIALS AND METHODS

### Cell culture

Human Umbilical Vein Endothelial Cells (HUVECs) were purchased from Lonza and maintained in Endothelial Cell Basal Medium (EBM-2) supplemented with 2% foetal calf serum (Sigma) and the following growth factors: 10 ng.mL^−1^ vascular endothelial growth factor, 5 ng.mL^−1^ human basic fibroblast growth factor, 1 μg.mL^−1^ hydrocortisone, 50 ng.ml^−1^ gentamicin, 50 ng.mL^−1^ amphotericin B, and 10 μg.mL^−1^ heparin. These growth factors were supplied as a bullet kit (Cell Media and Bullet Kit, Lonza).

CD31-positive human colorectal cancer liver metastases endothelial cells (CLMECs) and matched normal liver endothelial cells (LiECs) were isolated from patients undergoing curative liver resection for colorectal liver metastases at St. James's University Hospital, Leeds Teaching Hospitals NHS Trust. Patients provided fully informed, written consent. The work was carried out under approval granted by the Local Research Ethics Committee (Ref: 14/YH/1001). Tissue was taken immediately following surgical resection. A 2.5 cm × 2.5 cm × 2.5 cm incision was made into the tumour avoiding the centre and any obvious areas of necrosis. A separate sample of macroscopically normal liver was taken at least 2.5 cm away from the tumour. The tissue samples were stored in EBM-2 media on ice. Endothelial cells were isolated using a CD31 microbead technique. Initially tissue was minced using 2 scalpel blades and re-suspended in a dissociation solution consisting of 9 mL 0.1% Collagenase II, 1 mL 2.5 U.mL Dispase, 1 μM Calcium Chloride and 1 μM Magnesium Chloride in Hanks Buffer solution. The tissue-dissociation mix was incubated at 37°C for 45 minutes in a MACSMix Tube Rotator (Miltenyi Biotech) to provide continuous agitation. At the end of enzymatic digestion the sample was passed through 100 μm and 40 μm cell strainers to remove any undigested tissue. Cells were washed twice in magnetically-activated cell sorting (MACS) buffer consisting of Phosphate Buffered Saline (PBS), 2 mM EDTA and 0.1% Bovine Serum Albumin (BSA), pH 7.2. The washed pellets were suspended in 20 mL red blood cell lysis buffer consisting of 0.206 g Tris base, 0.749 g NH_4_Cl in 100 mL PBS pH to 7.2 for 10 minutes and then washed a final time in MACS buffer. Next the pellet was incubated with 200 μL of dead cell removal paramagnetic microbeads per 1 x10^7^ cells (Miltneyi Biotec) at room temperature for 15 minutes. After incubation the cells were passed through an LS column prepared with 1 × Binding Buffer (Miltenyi Biotec) in a magnetic field (MiniMACS Separator, Miltenyi Biotec). The eluate was then incubated with 30 μL FcR blocking reagent and 30 μL CD31-conjugated paramagnetic microbeads (Miltneyi Biotec) at 4°C for 15 minutes. After incubation this solution was passed through an MS column prepared with MACS buffer. CD31 positive cells were retained in the column and CD31 negative cells passed through as eluate. CD31 positive cells were washed through with warm EBM-2 media and placed in one well of a 0.1% gelatin coated 6-well plate and incubated in a 5% CO_2_ incubator at 37°C. Media was changed at 12 hrs and then every 48 hrs until confluent. Cells were used from passage 1-5.

### RNA isolation and RNA sequencing

Cells were grown to confluence in a 6-well culture plate and lysed with ice-cold TRI reagent. 12.5 μl of glycogen (RNA co-precipitant, Sigma) and 100 μl of bromochloropropane were added and the solution vortexed thoroughly before incubating for 15 min at room temperature. The sample was centrifuged at 13,000 rpm (4°C, 15 min) to separate the RNA, which was then precipitated with equal v/v ice-cold isopropanol. Following pelleting and washing with 75% ethanol the RNA was dissolved in 10 μl of nuclease free water before treatment with DNase I and quantification using Ribogreen.

RNA sequencing was performed using a TruSeq Stranded Total RNA Kit (Illumina) on an Illumina HiSeq 2000 next generation sequencer. RNA quality was confirmed using a bioanalyzer and universal human reference total RNA (Agilent). RNA samples were purified by removing ribosomal RNA using Ribo Zero rRNA magnetic removal beads (Illumina). RNA was fragmented and primed using divalent cations at 68°C and random hexamers. SuperScript II reverse transcriptase (Invitrogen) was used to synthesise first-strand cDNA. Second-strand synthesis removed the RNA template (RNaseH), replaced dTTP with dUTP and generated blunt-ended double-stranded cDNA (DNA Polymerase I). 3′ ends were adenylated to allow ligation of adapters for hybridization to a flow cell. Ligated cDNA fragments were then selectively amplified in a PCR reaction with primers targeted to the end of the successfully ligated adapters. Samples were then pooled for sequencing. The library was validated using a bioanalyzer to confirm the quality of the libraries prior to sequencing. Samples were run on one lane of the Illumina HiSeq 2000 using a paired-end sequencing strategy in which 100 bp reads were generated. Sequenced reads were analysed as described previously [18]. Briefly, sequenced reads were pre-processed to check read metrics, filter and then trimreads. Reads were aligned to the human reference genome version 19 using Tophat. Multireads were assigned using the programme SEQEM [19]. Data were expressed as counts per million: 10^6^. Z.Cx/lN, where Z is the normalization factor (mean length of expressed transcripts), Cx the number of reads mapping to transcript x, l the length of transcript x, and N the total number of mappable reads.

### RNA interference screen

Reverse transfection of HUVECs was undertaken on a 96-well plate using Dharmacon siGenome siRNA (50 nM per well), 0.1 μL RNAiMAX and 5000 cells per well. Cells were incubated for 72 hrs and then stained with Hoescht (1:1000), before being exposed to 3 μM PGPC for 10 minutes. Cells were then fixed, imaged and analysed with plate-wise robust Z scores (sample median – plate median / median absolute deviation). In a second validation screen reverse transfection was undertaken on a 96-well plate using Dharmacon On-Targetplus siRNA (50 nM per well) with other steps remaining unchanged.

### WEE1 transfection

Knockdown of WEE1 in endothelial cells was achieved using a transient transfection method. Cells were transfected once 90% confluent. For transfection of one well of a 6 well plate, 200 μL of Opti-MEM containing 2% Lipofectamine 2000 was added to 200 μL Opti-MEM containing 250 nM Dharmacon On-Targetplus pooled WEE1 siRNA and left at room temperature for 20 minutes. This 400 μL transfection mix was added to 600 μL fresh EBM-2 media and added to the well, giving a final WEE1 siRNA concentration of 50 nM. This was applied for 3 hrs and incubated in a 5% CO_2_ incubator at 37°C. After 3 hrs the transfection mix was removed and replaced with fresh EBM-2 media. Cells were incubated for a further 24 hrs before being used in experiments. WEE1 knockdown was confirmed with Western blot.

### Western blotting

Cells were harvested in lysis buffer containing 10 mM Tris, pH 7.5, 150 mM NaCl, 0.5 mM EDTA, 0.5 % NP-40, MiniComplete protease inhibitors (Roche), and PhosSTOP phosphatase inhibitors (Roche). Equal protein amounts were loaded on 4-20% gradient gels and resolved by electrophoresis. Samples were transferred to PVDF membranes and labelled overnight with primary antibody: mouse anti-human β-Actin (1:2000, Santa Cruz Biotechnology), mouse anti-human WEE1 B-11 (1:300, Santa Cruz Biotechnology), mouse anti-human CD31 (1:1000, Dako, Clone JCT0A), rabbit anti-human pCDK1-Y15 (1:1000, Cell Signaling Technology), mouse anti-human CDK1 (1:1000, Cell Signaling Technology). Species appropriate secondary antibodies and SuperSignal Femto detection reagent (Perbio Science) were used for visualisation.

### Immunofluorescence

Cells were fixed in 4% paraformaldehyde and permeablised with 0.1% TritonX-100 at room temperature. Cells were blocked with donkey serum for 30 minutes to prevent non-specific binding. Cells were then incubated with 1% BSA in PBS containing the following primary antibody dilutions for 1 hr at room temperature; mouse anti-human CD31 (1:300, Dako, Clone JCT0A), mouse anti-human vWF (1:100, Dako), rabbit anti-human VE-Cadherin (1:200, Cell Signaling Technology), rabbit anti-human VEGFR-2 (1:100, Cell Signaling), mouse anti-human eNOS (1:100, BD Biosciences). Following incubation with primary antibody, cells were incubated with species appropriate, fluorophore tagged secondary antibodies for 45 minutes at room temperature. Cells were mounted with Prolong Gold Antifade Reagent (Invitrogen) and visualised using a LSM 880 confocal microscope (Zeiss).

### Intracellular Ca^2+^ measurement in response to VEGF

Intracellular Ca^2+^ was measured using the ratiometric Ca^2+^ indicator dye, Fura-2. CLMECs were grown to confluence in a 96-well plate overnight. The following morning media was removed and cells in each well were incubated with 50 μL Fura-2 loading solution for 1 hr at 37°C. The Fura-2 loading solution consisted of 2 μM Fura-2 AM and 0.01% Pluronic Acid in Standard Bath Solution (Ca^2+^-SBS) consisting of 130 mM NaCl, 5 mM KCl, 1.2 mM MgCl_2_, 1.5 mM CaCl_2_, 8 mM D-Glucose and 10 mM HEPES. After 1 hr the loading solution was removed and 200 μL of Ca^2+^-SBS was added to each well and left at room temperature for 10 minutes. A compound plate was prepared containing Vascular Endothelial Growth Factor (VEGF, Sigma) at five times the final concentration (150 ng.mL^−1^) in Ca^2+^-SBS. The FlexStation II^384^ was set to add 50 μL of the VEGF compound solution to each well on the test plate containing 200 μL of Ca^2+^-SBS. This gave a final VEGF concentration of 30 ng.mL^−1^, which reflects physiological levels. Baseline fluorescence ratios were recorded before addition of the compound solution to the cell plate after 60 seconds, with regular recordings thereafter for a total of 10 minutes.

### Alignment studies

CLMECs were grown to 80% confluence in a 6-well plate. Shear stress was achieved by placing the cells on an orbital shaker for 48 hrs (153 rpm). The swirling motion of the media around the edge of the wells produces tangential shear stress, resulting in cell elongation and alignment. After 48 hrs cells were imaged and analysed using ImageJ software to determine the degree of alignment. To do this, images were rotated to the direction of applied shear stress and were processed using a Difference of Gaussian plugin to define cell edges (http://www.sussex.ac.uk/gdsc/intranet/microscopy/imagej/utility). Quantification of cell orientation relative to the direction of shear stress was determined using OrientationJ software (http://bigwww.epfl.ch/demo/orientation/). OrientationJ generates a histogram of all local angles in each image and a Gaussian distribution curve was fitted. From this, the baseline-subtracted frequency maximum at the mode of the distribution was determined.

### WST-1 proliferation assay

WST-1 is a colorimetric assay used for the non-radioactive quantification of cell proliferation, viability and cytotoxicity. Cells were plated onto a 96-well plate and grown overnight. The following morning cells were drugged and grown for 48 hrs with a drug change at 24 hrs. Upon completion of the experiment WST-1 was applied in accordance with the manufacturer's protocol and absorbance was measured in a microtiter plate reader at wavelengths between 440 and 650 nm.

### Migration assay

Cell migration was measured using the Incucyte™ FLR Kinetic Imaging System (Essen). Cells were plated onto a 96-well plate (Essen Imagelock, Essen) and grown overnight to confluence. The following morning a linear scratch (wound) was made in every well using the Essen Woundmaker™ (Essen). Following the scratch, cells were drugged before being placed in the Incucyte™ FLR Kinetic Imaging System. Cells were imaged every hour for 24 hrs or until they had fully migrated across the scratch wound. Migration was measured automatically by the Incucyte™ FLR Kinetic Imaging System, which calculates Relative Wound Density (RWD). This measures the spatial density in the scratch wound relative to the spatial density of cells outside of the scratch wound. It is 0% at 0 hrs and reaches 100% once the spatial density inside the scratch is the same as outside. This allows data to be self-normalised against changes in cell density that occur outside of the wound due to cell proliferation.

### Matrigel® tube formation assay

To assess Matrigel® induced tube formation, 50 μL of 10 mg.mL^−1^ Matrigel® (Corning) was plated in each well of a 96-well plate and incubated overnight. The following morning 20,000 CLMECs were plated on top of the Matrigel® in each well and grew into tubular structures within 24 hrs. Phase-contrast images were obtained using an Incucyte™ FLR Kinetic Imaging System and the number of loops were counted using ImageJ software.

### Co-culture tube formation assay

*In vitro* tube formation was studied using an endothelial/fibroblast co-culture assay. Normal Human Dermal Fibroblasts (NHDF, Lonza) were seeded at 6,000 cells per well in a 96-well plate (Greiner Bio-one) and allowed to grow to a confluent monolayer over 4 days. On day 5 endothelial cells were seeded on top of the fibroblast monolayer at 6,000 cells per well and allowed to grow overnight. Over the next 5 days endothelial cells reliably grew into tube structures and were treated daily with either AZD1775 (1 μM) or vehicle control (days 6-10). On day 11 cells were stained for CD31 to assess tube formation. Firstly, cells were fixed in 4% paraformaldehyde and permeabilised with 0.1% TritonX-100 at room temperature. After three washes in PBS the cells were blocked in donkey serum for 30 minutes at 37°C. Cells were then incubated with 1% BSA in PBS containing mouse anti-human CD31 (Dako, Clone JCT0A) at 1:300 dilution for 1 hr at 37°C. Following washing, cells were incubated with Alexa 488-conjugated Affinipure Donkey anti Mouse IgG (Jackson Immuno Research Laboratories) at 1:300 dilution for 45 minutes at 37°C. Cells were then incubated with 100 μL PBS and imaged on the Incucyte™ FLR Kinetic Imaging System in phase-contrast and fluorescence mode using a x10 objective. Tube length, number of branch points and tube surface area were calculated using inbuilt algorithms.

### Apoptosis assay

CLMEC apoptosis was measured using a caspase-3 assay on the Incucyte™ FLR Kinetic Imaging System. CLMECs were plated onto a 6-well plate and grown overnight. The following morning cells were treated with 5 μM NucView™488 caspase-3 substrate (Biotium) according to manufacturer's instructions in the presence of AZD1775 (1 μM) or vehicle control. Cells were placed in the Incucyte™ FLR and imaged every hour for 24 hrs in phase contrast and fluorescence mode using a x10 objective. After 24 hrs cell nuclei were stained with 5 μM Vybrant® Dye Cycle™ (green) (Molecular Probes, Invitrogen). Apoptotic Index was calculated as the percentage of caspase-3 positive cells divided by the total number of cells.

### Flow cytometry

All flow cytometry work was undertaken on a BD-LSR Fortessa Flow Cytometer. Endothelial cells were plated at 300,000 cells per well on a 6-well plate and grown overnight. The following morning cells were treated and after 24 hrs were trypsinised and spun down. Ice-cold ethanol (70%) was added dropwise to each pellet before samples were frozen at -20°C for at least 48 hrs. To detect double-stranded DNA breaks, samples were defrosted, washed twice with PBS and then incubated with Alexa Fluor 488 Mouse anti-γH2AX (BD Biosciences) for 20 minutes at room temperature. Following incubation samples were washed again with PBS before adding 0.5 mL per 1 × 10^6^ cells PI/RNase Buffer (BD Biosciences). Samples were incubated at room temperature for 15 minutes before being analysed on the flow cytometer. All analysis was undertaken using BD FACSDiva v6.2 software.

### Data analysis

Statistical Analysis was performed using OriginPro 9.1 (OriginLab Corporation). Data are presented as mean +/− SEM. Prior to statistical analysis, data were tested for normality and equality of variance. Paired data were compared statistically using t tests. For data sets with more than 2 groups ANOVA and a post-hoc Bonferroni test were used. “n” indicates the number of independent experiments, where as “N” indicates the number of replicates. For statistical significance * indicates p<0.05, ** p<0.01 and ***p<0.001.

## SUPPLEMENTARY MATERIALS FIGURES AND TABLES






