# Supplementary material for: Upregulated WEE1 protects endothelial cells of colorectal cancer liver metastases
Source: Oncotarget. 2017 Feb 2;8(26):42288–99. doi: 10.18632/oncotarget.15039 (PMC5522067; doi:10.18632/oncotarget.15039)
Supplement: Supplementary file 1 [file oncotarget-08-42288-s001.pdf]

Upregulated WEE1 protects endothelial cells of colorectal cancer liver metastases

SUPPLEMENTARY MATERIALS

Sex	Age (yrs)	Primary Colorectal Cancer	Colorectal Liver Metastases	Histology of Colorectal Liver Metastases	Medical History	Drug History	Social History
F	85	Rectal Adenocarcinoma pT2 N0 V0 M0 R0<br><br>Moderately differentiated Dukes Stage B	Metachronous 16 months post primary<br><br>4 lesions in segments 6,7 and 8	Moderately differentiated adenocarcinoma R0	Asthma Osteoarthritis Hypothyroidism	Levothyroxine, Dosulepin, Fybogel	Non-smoker, 5 units alcohol/ week
F	70	Caecal Adenocarcinoma pT4 N0 V0 M0 R0<br><br>Moderately differentiated Dukes Stage B	Metachronous 12 months post primary<br><br>1 lesion in segment 4b/5	Moderately differentiated adenocarcinoma R0	Chronic Kidney Disease Stage II, Osteoarthritis, Hiatus Hernia	Paracetamol, Fentanyl, Quinine Sulphate, Bisoprolol, Movicol, Amitryptiline	Non-smoker, 5-10 units alcohol/week
M	75	Rectal Adenocarcinoma pT2 N0 V0 M0 R0<br><br>Well differentiated Dukes Stage B	Metachronous 48 months post primary<br><br>1 lesion in segment 1	Moderately differentiated adenocarcinoma R0	Ischaemic Heart Disease, Benign Prostatic Hypertrophy	Aspirin, Bisoprolol, Ramipril, Simvastatin	Non-smoker, No alcohol

Supplementary Figure 1: Patient characteristics of samples used for RNA sequencing.

Gene Code	Protein	Function	Mean CLMEC Counts per Million	Mean LiEC Counts per Million	CLMEC/LiEC Ratio	CLMEC vs. LiEC p value
CEACAM5	Carcinoembryonic Antigen Related Cell Adhesion Molecule 5	Cell Adhesion, Cancer Biomarker	298.09	0.03	8792.02	6.22 E-76
EPCAM	Epithelial cell adhesion molecule	Cell Adhesion, Cancer Biomarker	162.54	0.39	407.93	2.13 E-45
CEACAM6	Carcinoembryonic Antigen Related Cell Adhesion Molecule 6	Cell Adhesion, Cancer Biomarker	81.62	0.75	108.16	1.41 E-33
CFTR	Cystic Fibrosis Transmembrane Conductance Regulator	Ion Transport	122.12	1.18	103.20	4.92 E-21
FXYD3	FXYD domain-containing ion transport regulator 3	Ion Transport	14.49	0.21	69.50	5.86 E-19
FAT1	Protocadherin FAT1	Tumour Suppressor Gene, WNT-signalling	141.27	4.18	33.80	1.15 E-17
RAB25	Ras-related protein Rab-25	Proto-oncogene	8.08	0.13	63.31	4.85 E-14
LGR5	Leucine Rich Repeat Containing G Protein-Coupled Receptor 5	WNT-signalling	37.45	0.33	114.53	2.16 E-13
MMP7	Matrix Metalloproteinase-7	Extracellular Matrix Degradation, Invasion, Angiogenesis	20.17	0.26	78.97	1.13 E-11
ANTXR1 (TEM8)	Anthrax Toxin Receptor 1	Tumour Endothelial Marker	10.79	3.5	3.08	0.035

Supplementary Figure 2: Selection of upregulated transcripts of potential interest in colorectal cancer and angiogenesis.

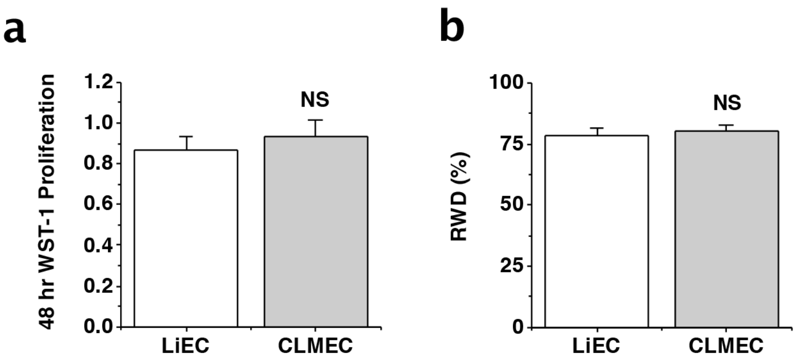

Supplementary Figure 3: Comparison of matched CLMEC and LiEC functional characteristics. a. Mean data for CLMEC and LiEC proliferation at 48 hrs measured using WST-1 reagent (n=5, N=15 each). **b.** Mean data from the relative wound density (RWD) in matched CLMECs and LiECs, 15 hrs after making a scratch wound (n=3, N=9 each).

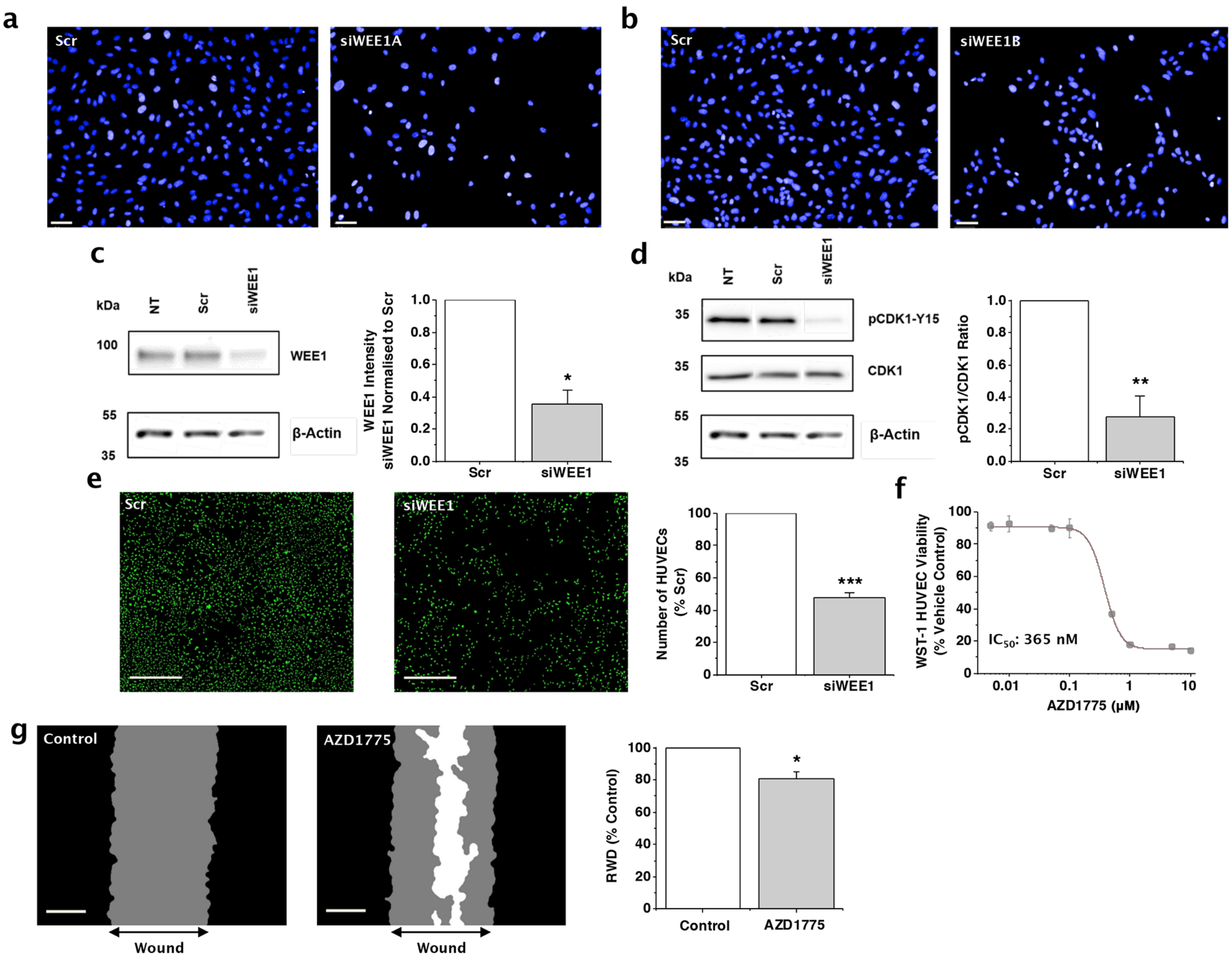

Supplementary Figure 4: WEE1 has functional significance in HUVECs. a. Example images of Hoechst staining of HUVECs 72 hrs after transfection with scrambled siRNA (Scr) or with siGENOME WEE1 siRNA (siWEE1A) as part of an RNA interference initial screen. Scale bars 50 µm. **b.** Further example images validating the RNA interference initial screen findings in (a). Here, ON-TARGETplus WEE1 siRNA (siWEE1B) has been used. Scale bars 50 µm. **c.** On the left, Western blot labelled with anti-WEE1 and anti-β-Actin antibodies for non-transfected (NT) HUVECs and HUVECs transfected with scrambled siRNA (Scr) or ON-TARGETplus WEE1-targeted siRNA (siWEE1). On the right, quantification of the WEE1 band intensity for the siWEE1 group normalised to the Scr group (n=3 each). **d.** On the left, Western blot labelled with anti-pCDK1-Y15, anti-CDK1 and anti-β-Actin antibodies for non-transfected (NT) HUVECs and HUVECs transfected with scrambled siRNA (Scr) or WEE1-targeted siRNA (siWEE1). On the right, quantification of the pCDK1-Y15 band intensity divided by the CDK1 intensity (n=3 each). **e.** On the left, fluorescence images of HUVECs 48 hr after transfection with scrambled (Scr) or WEE1 siRNA (siWEE1). Fluorescence was from cell nuclei stained with Vybrant® Dye Cycle™ (green). Scale bars 400 µm. On the right, normalised quantification of the images from independent repeats (n=3, N=18). **f.** Mean data for HUVEC viability measured using WST-1 reagent after treatment with AZD1775 at the indicated concentrations and plotted as percentages of the vehicle control (n=3, N=9). **g.** On the left, example linear wound mask images after 24 hr HUVEC migration in vehicle control (Control) or 1 µM AZD1775. Black represents cells outside the linear wound (top to bottom), grey - cells which had migrated into the wound, and white - no cells. Scale bars 200 µm. On the right, mean data from images of the type shown on the left. Relative wound density (RWD) in the presence of AZD1775 is presented as a percentage of Control (n=3, N=12).

Supplementary Data File 1: RNA sequencing of matched CLMEC and LiEC data set.

See Supplementary Data File 1

Supplementary Data File 2: RNA interference screen and validation of hits in HUVECs.

See Supplementary Data File 2
